# Addition of metformin to anti-PD-1/PD-L1 drugs activates anti-tumor immune response in peripheral immune cells of NSCLC patients

**DOI:** 10.1038/s41419-025-07636-7

**Published:** 2025-04-13

**Authors:** Luisa Amato, Caterina De Rosa, Gaetano Di Guida, Filippo Sepe, Annalisa Ariano, Sara Capaldo, Faiz Ul Haq, Alessandra Di Liello, Concetta Tuccillo, Stefano Lucà, Renato Franco, Viviana De Rosa, Francesca Iommelli, Alberto Servetto, Fortunato Ciardiello, Carminia Maria Della Corte, Floriana Morgillo

**Affiliations:** 1 Department of Precision Medicine, University of Campania Luigi Vanvitelli, 80131 Naples, Italy; 2 Pathology Unit, Department of Mental and Physical Health and Preventive Medicine, University of Campania “Luigi Vanvitelli”, 80138 Naples, Italy; 3 Institute of Biostructures and Bioimaging, National Research Council, 80145 Naples, Italy; 4 Department of Clinical Medicine and Surgery, University of Naples Federico II, Naples, Italy

**Keywords:** Translational immunology, Non-small-cell lung cancer, Tumour immunology, Cancer immunotherapy

## Abstract

Immunotherapy has transformed the treatment landscape for non-small cell lung cancer (NSCLC), yet achieving lasting benefits remains a challenge. The resistance mechanisms to immunotherapy are complex, involving interactions between tumor cells and immune cells that are not fully understood. Metformin, an FDA-approved diabetes medication, shows promise in enhancing immunotherapy efficacy by boosting anti-tumor immune responses, although the underlying molecular pathways are still being investigated. This study utilized co-culture models of cancer and immune cells to explore the effects of combining metformin with anti-PD-1/PD-L1 therapies on the anti-tumor immune response in LKB1 mutant (LKB1mut) versus wild-type (LKB1wt) NSCLC cells, alongside peripheral blood immune cells from NSCLC patients. The transcriptomic profiles of LKB1mut and LKB1wt NSCLC cells were characterized via bulk RNA sequencing to understand gene expression changes induced by metformin. Patients with advanced-stage NSCLC provided peripheral blood mononuclear cells (PBMCs) for analysis. The study assessed metformin’s impact both alone and in combination with anti-PD-1/PD-L1 agents on innate immune pathways. Results indicated that metformin activated the cGAS-STING pathway and interferons in PBMCs, enhancing their anti-tumor capabilities. Notably, immune cells treated with metformin and immunotherapy exhibited synergistic effects, significantly reducing colony formation in LKB1mut NSCLC cells. Additionally, monocytes from NSCLC patients showed decreased viability of NSCLC cells in co-culture, independent of LKB1 status, and shifted towards an anti-tumor M1 phenotype with combined treatment. These findings were supported by 3D co-culture models involving tumor spheroids and patient-derived organoids, highlighting a novel biological rationale for using metformin alongside immunotherapeutic agents to boost anti-tumor activity across various immune cell subsets derived from NSCLC patients.

## Introduction

Lung cancer is one of the most frequently diagnosed cancers worldwide and is the leading cause of cancer-related mortality [1]. Non-small cell lung carcinoma (NSCLC) accounts for the majority of cases (85%) and comprises a heterogeneous group that includes lung adenocarcinoma (LUAD), lung squamous cell carcinoma (LUSC), and large cell carcinoma (LCC) [2]. Although the advent of anti PD-1/PD-L1 drugs has revolutionized the management of patients with advanced NSCLC, durable benefits are not achieved in all patients [3], and the mechanisms of resistance remain largely unknown.

In LUAD, serine/threonine kinase 11 (STK11), also known as liver kinase B1 (LKB1), ranks as the third most commonly mutated gene after TP53 and KRAS, with mutations found in up to 33% of primary LUAD cases [4]. Tumors harboring STK11 mutations exhibit an “immune-cold” phenotype characterized by low or absent PD-L1 levels and ineffective T cell infiltration. Additionally, STK11/LKB1 inactivation is a major genetic driver of primary resistance to anti-PD-1 agents and co-mutation of STK11 and KEAP1 genes has been correlated to higher sensitivity to dual immunotherapy with anti-CTLA4 and anti-PD-1 agents versus anti-PD1 alone [5, 6]. In this context, our group previously demonstrated that stimulators of interferon genes (STING) activation identifies NSCLC patients with an immune-responsive phenotype [7]. Mechanistically, STK11 mutations lead to LKB1 loss, which results in the suppression of (STING). Thus, reactivating STING in STK11 mutant LUAD has been proposed as a promising approach to support anti-tumor immune response [8]. Recently, we also established that cGAS-STING activation in peripheral blood mononuclear cells (PBMCs) from lung cancer patients, particularly those with NSCLC, may serve as a novel predictive biomarker for response to immunotherapy [9].

Several evidences have suggested that the anti-diabetes drug metformin (MET) exerted anti-cancer effects in various cancer types, including lung, prostate, and colon [10]. Preclinical data support the role of MET as an adjuvant drug in the treatment of lung cancer, both in combination with chemotherapy and with targeted drugs and immunotherapy [11]. In particular, MET may modulate different components of the immune-microenvironment and promote pro-immune features [12]; however, it is unknown whether MET may enhance the efficacy of immunotherapy in particular in STK11 mutant NSCLC.

Our phase I/II dose-escalation study evaluated the feasibility of metformin treatment also in a non-diabetic population of cancer-patients and in particular confirmed the acceptable safety profile of the combination of metformin and erlotinib as a second-line treatment for stage IV NSCLC with wild-type EGFR [13, 14], making it an ideal partner drug for novel combinations.

In this study, we hypothesized that MET treatment may impact on activation of anti-tumor immune response, including STING activation and related pathways, with a direct effect in immune cells. Here, we sought to assess the efficacy of combining MET with immunotherapeutic agents in LKB1-mutant NSCLC tumors and in various subsets of PBMCs, specifically lymphocytes and monocytes isolated from treatment-naive NSCLC patients. Finally, we validated the in vitro and ex vivo findings in two-dimensional and three-dimensional co-culture models utilizing tumor spheroids and patient-derived tumor organoids (PDTOs) to elucidate the impact of metformin and anti-PD-1/PD-L1 immunotherapy on the interactions between cancer and immune cells.

## Materials and methods

### Reagents

Metformin, Atezolizumab, and Pembrolizumab, were purchased from Selleck Chemicals (Houston, TX).

### Cell lines

The NSCLC cell lines NCI-H1299 (ATCC Cat#CRL-5803) and NCI-H460 (ATCC Cat#HTB-177) were maintained in RPMI-1640 (Sigma-Aldrich) supplemented with 10% FBS (Sigma-Aldrich), 1X penicillin–streptomycin (Sigma-Aldrich) and 1X Amphotericin B (Sigma-Aldrich) in a humidity-controlled environment (37 °C, 5% CO_2_). Cell lines were obtained from the American Type Culture Collection (ATCC). Cell lines morphology was monitored, and the cell lines were routinely tested for mycoplasma using a mycoplasma detection kit (InvivoGen).

### RNA-sequencing

Cell lines and PBMCs were treated with 2 mM Metformin and 10 μg/ml Atezolizumab for 48 h. Cells were harvested and RNA was purified using an RNeasy Plus Mini kit (QIAGEN). The samples were analysed by Novogene Co., Ltd. Detailed RNA-seq procedure is described in the Supplemental Material file.

### MTT assay

MTT assay was performed as previously described [15]. Briefly, cells were seeded in 96-well plates at a density of 5000 cells/well and co-cultured with PBMCs (ratio 1:10). After 48 h of treatment, the methylthiazolyldiphenyl-tetrazolium bromide (MTT, Sigma Chemical Co.) was added. After 4 h, cells were lysed by addition of DMSO. The number of viable cells was determined spectrophotometrically by measuring absorbance at 490 nm.

### Western blot analysis

Protein lysates were obtained by homogenization in RIPA buffer [0.1% SDS, 0.5% deoxycholate, 1% Nonidet, 100 mmol/L NaCl, 10 mmol/L Tris–HCl (pH 7.4), 0.5 mmol/L DTT, 0.5% PMSF, protease inhibitor cocktail (Hoffmann-La Roche), and PhosSTOP (Roche Diagnostics)] and clarified by centrifugation at 12.450 rpm for 20 min at 4 °C. Protein amounts were estimated by a modified Bradford assay (Bio-Rad), resolved by SDS-PAGE and electrotransferred onto nitrocellulose membranes (Bio-Rad). After blocking the membranes for 90 min at RT, they were incubated overnight at 4 °C with primary antibodies, followed by incubation with a secondary antibody for 1 h at RT. Proteins were detected with Clarity Western ECL Substrate using a ChemiDoc (Bio-Rad). Images were analyzed using Image Lab 3.0.1. Primary antibodies for western blot analysis against LAMIN A/C (2032, 1:1000), CASPASE-8 (1C12) (9746, 1:1000), and β-Actin (8H10D10, 1:2000) was purchased from Cell Signaling.

### Patients cohort and PBMCs isolation

Patients with a diagnosis of NSCLC at baseline before receiving any treatment were enrolled. The patients’ characteristics are listed in Table 1. Representative IHC images of PD-L1 tumor expression levels in tissue biopsies are reported in Supplementary Fig. S1. Blood samples were collected after obtaining a written informed consent from patients in accordance with the Declaration of Helsinki (protocol approved by the Ethics Committee of the University of Campania “Luigi Vanvitelli” and Naples No. 280 on May 16, 2020). NSCLC patients’ blood was collected in BD vacutainer spray-coated K2EDTA tubes as previously described [9]. Briefly, PBMCs were isolated by Lymphosep (Aurogene). PBMCs were washed with PBS and cultured in RPMI-1640 medium (Sigma-Aldrich) supplemented 10% FBS, and 1X penicillin–streptomycin (Sigma-Aldrich). After 24 h, the monocytes were mechanically isolated by adherence, and lymphocytes were separated by suspension.

**Table 1:** Baseline clinical characteristics of patients.

Variable	Patients^a^ (*n* = 18)
**Age, years (mean** **±** **SD)**	**70.56** ± **7.21**
**Sex (*n*, %)**	
Male	**9 (56.25%)**
Female	**7 (43.75%)**
**Clinical stage (*n*, %)**	
III	**2 (12.5%)**
IV	**14 (87.5%)**
**PD-L1 expression** (***n*, %)**	
< 1%	**9 (56.25%%)**
> 1%	**7 (43.75%%)**

Data are reported as number (*n*), frequency (%) or mean ± SD: standard deviation. ^a^Patients race/ethnicity was White/Italian.

### Immunohistochemistry (IHC)

Immunohistochemistry (IHC) was performed on 4-micron sections of formalin-fixed, paraffin-embedded (FFPE) tissue samples using Ventana platform (Ventana BenchMark ULTRA system), according to the manufacturer’s instructions. The following antibody was used: anti-PD-L1 (rabbit monoclonal primary antibody, clone SP263, VENTANA). The IHC staining were interpreted based on the percentage of tumor cells (TCs) exhibiting membranous staining at any intensity and it was scored according to the IMpower010 study data [16]:

- Negative staining: PD-L1 expression on <1% of TCs;
- Positive staining: PD-L1 expression on ≥1% of TCs;

The PD-L1 positive cases were stratified based on the percentage of positive TCs:

- PD-L1 expression ranging from 1 to 49% of TCs;
- PD-L1 expression on ≥50% of TCs.

### Colony formation assay

1000 cells/well were seeded on a 6-well plate. The next day the plates were treated with 2 mM Metformin, 10 μg/ml Atezolizumab and 10 μg/ml Pembrolizumab. After 10 days, colonies were fixed with 4% PFA and stained with 1% crystal violet in 10% methanol.

### RNA extraction, cDNA synthesis and qRT-PCR

Total RNA was obtained using TRIzol (Invitrogen), according to the manufacturer’s protocol. Briefly, cells were lysed with TRIzol. Chloroform was added and samples were then centrifuged at 12,000 x g for 15 min at 4 °C. The aqueous phase was transferred to another tube. RNA was precipitated by mixing with isopropyl alcohol for 2 h at −80 °C. Samples were centrifuged at 12,000 x g for 10 min at 4 °C and washed with 75% ethanol. Total RNA was suspended in RNase-free water, 0.1 mM EDTA solution and incubated in a heat block at 57.5 °C for 10 min. The purity and concentration of RNA were determined by OD 260/280 readings using the Nanodrop 2000 spectrophotometer (Thermo Fisher Scientific). cDNA was generated using a SensiFAST cDNA Synthesis Kit (Meridian Bioscience). mRNA expression levels were evaluated by qRT-PCR with a QuantStudio 7-Flex (Applied Biosystems) using the SensiFAST SYBR Hi-ROX Kit (Meridian Bioscience). Relative gene expression was determined by normalizing to 18S and calculated by the 2-ΔΔCt method. The list of primer sequences used for qRT-PCR are shown in Supplementary Table 1.

### Spheroids formation and Immunofluorescence staining

Tumor spheroids were generated by seeding 8 × 10^4^ cells in ultra-low attachment (Corning) as previously described [9]. Co-cultures were initiated by adding 5 × 10^5^ SCLC patients derived immune cells per well. Before co-culture, 2.5 µM CellTracker Deep Red dye (Invitrogen) was added to PBMC culture medium for 45 min and 5 µM CellTracker Blue CMAC (Invitrogen) was added to the spheroid culture medium for 45 min. After the staining, the PBMCs were added to spheroids (ratio 1:10) and the co-cultures were performed for 48 h. The fluorescence was imaged using a Cell Discoverer 7 microscope (ZEISS Olympus, Nikon). The quantification of fluorescence intensity was performed with ImageJ Software.

### Patient-derived tumor organoids generation

Tumor tissue obtained from a patient with NSCLC was utilized for the generation of tumor organoids as previously described [17]. The biopsy was processed by cutting it into small pieces of 2-4 mm and washed with PBS supplemented with 1% penicillin-streptomycin. The minced tissue was incubated with Digestion Medium (5 mg/ml Collagenase type II, 100 μg/ml DNase I, 10 μg/ml Y-27632 dihydrochloride in Advanced DMEM/F-12, 1X penicillin–streptomycin and 1X Amphotericin B) for 1 h at 37 °C on a shaking platform. The digested tissue was centrifuged at 200 *g* for 5 min at RT, washed with AdDMEM/F-12 and incubated with trypsin + Y-27632 dihydrochloride (10 μM) at 37 °C on a shaking platform. Then, cold adDMEM/F12 + ++ supplemented with 1X penicillin–streptomycin, 1X Amphotericin B and 20% FBS were added to stop the digestion. The resulting cells were cultured in Matrigel droplets of organoid culture medium (composition of organoid medium is listed in Supplemental Material).

### Flow cytometry analysis

Tumor-infiltrating lymphocytes were separated by Tumor cell isolation kit (Miltenyi Biotec) analyzed by flow cytometry. TILs staining: Live Dead Blue (Invitrogen), antibodies used were anti-human CD3 (UCHT1, SK7), CD4 (L3T4, SK3), CD8 (SK1), CD56 (NCAM16.2).

### Statistical analysis

Results are expressed as the mean ± SEM or SD. Two-group analyses were performed using an unpaired t-test. Three or more groups with one independent variable were analyzed using a one-way ANOVA test. Three or more groups with two independent variables were analyzed using a two-way ANOVA test. The Pearson correlation coefficients and the corresponding *p*-values were calculated using Prism 8 (GraphPad Software).

## Results

### Transcriptomic characterization of LKB1mut and LKB1wt NSCLC cells

**Fig. 1:**
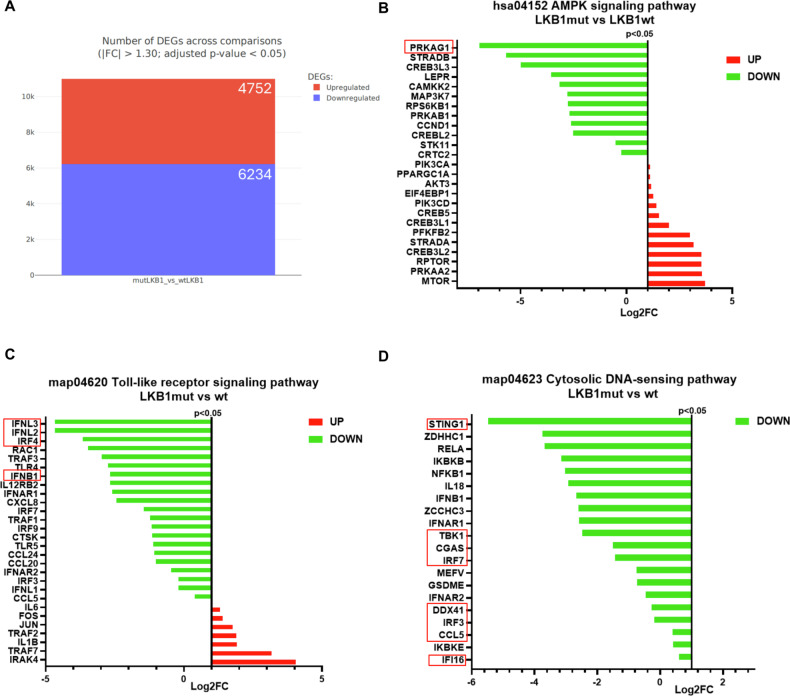
RNA-sequencing of LKB1mut vs LKB1wt NSCLC cell lines. **A** DEGs across LKB1mut vs LKB1wt comparisons. Fold Change (FC) > 1.30 and *p* value < 0.05. **B–D** Results of KEGG pathway enrichment analysis. The red and green bars represent the upregulated genes and downregulated genes, respectively for the differential expression of the selected pathways based on their RNA-seq expression. Data are presented as Log2FC values, all the listed genes *p* value is < 0.05.

We analyzed the transcriptomic features of LKB1-mutant NSCLC cells (H460) compared to LKB1-wild type one (H1299). As shown in Fig. 1A we found 4752 upregulated and 6234 downregulated genes (fold change (FC) > 1.30; *p* value < 0.05) in LKBmut cells compared to LKB1wt. Given the key role of LKB1 loss in the downregulation of AMPK activation, we analyzed AMPK signaling pathway (hsa04152) (Fig. 1B). RNA-seq data confirmed a downregulation of AMPK gene transcription (PRKAG1) and an upregulation of its signaling pathway along with mTOR mRNA upregulation in LKB1mut cells. Then, we wondered whether the LKB1 loss occurred along with a modulation of immune responsiveness in the selected cells. Interestingly, both the Toll-like receptor signaling pathway (map04620) (Fig. 1C) and the cytosolic DNA-sensing pathway (map04623) (Fig. 1D) showed a strong reduction of key genes transcription involved in immune response, such as IFNs, IRFs, TBK1 and cGAS-STING.

### Effect of metformin on cGAS-STING downstream pathway activation in LKB1mut and LKB1wt NSCLC cells

**Fig. 2:**
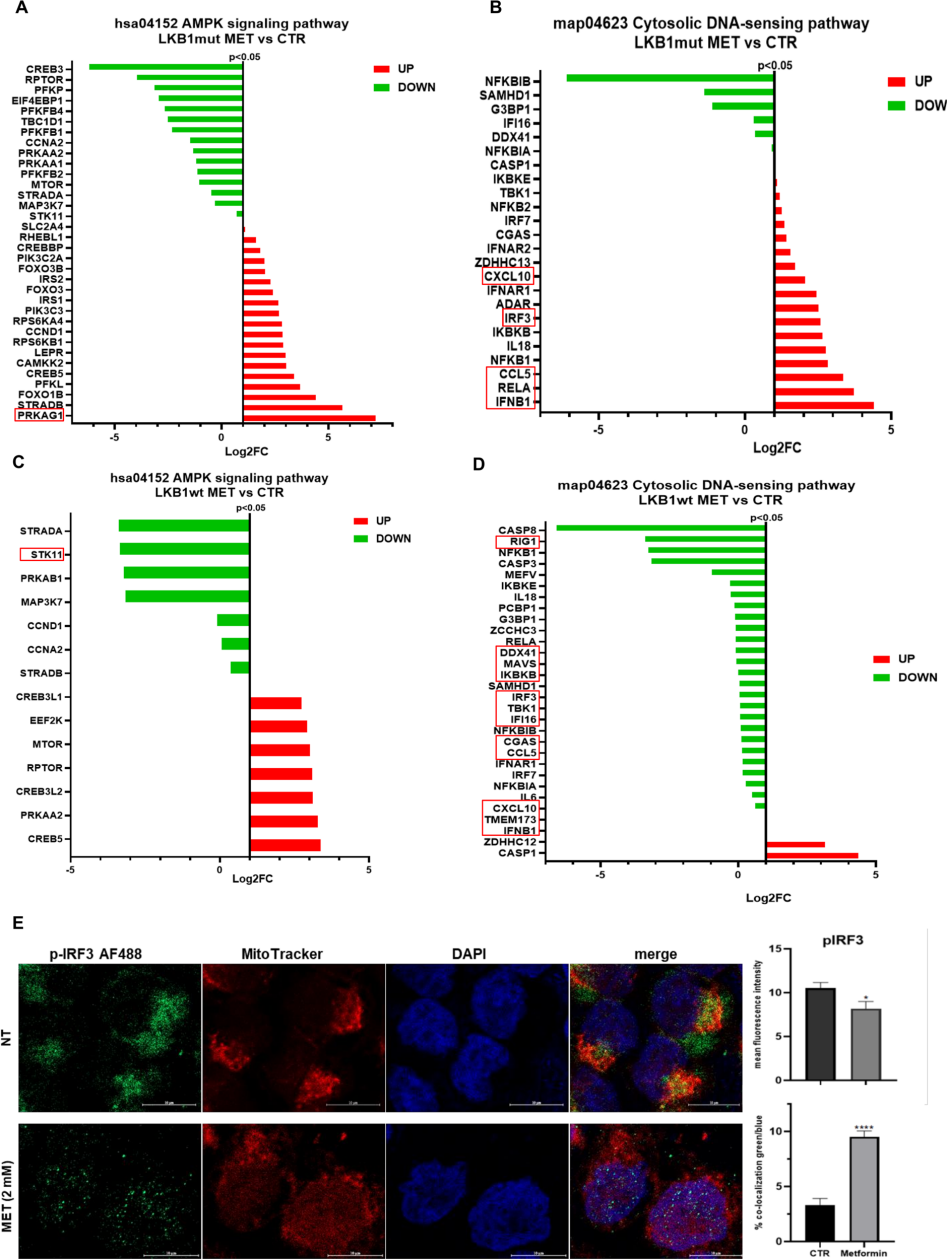
Effect of treatment with metformin on cGAS-STING downstream activation in LKB1mut NSCLC cell lines. **A, B** Results of KEGG pathway enrichment analysis in LKB1mut cells treated with MET compared to CTR. Data are presented as Log2FC values, all the listed genes *p* value is < 0.05. **C, D** Results of KEGG pathway enrichment analysis in LKB1wt cells treated with MET compared to CTR. The red and green bars represent the upregulated genes and downregulated genes, respectively for the differential expression of the selected pathways based on their RNA-seq expression. Data are presented as Log2FC values; all the listed genes *p* value is < 0.05. **E** Representative immunofluorescence images, p-IRF3 mean fluorescence intensity (MFI) and nuclear localization (green/blue merge) showing p-IRF3 (green), MitoTracker (red) and DAPI (blue) in H460 cells treated with MET 2 mM (scale bar: 10 μm, Magnification 63X). Each experiment was performed in three technical replicates. Data are expressed as mean ± SD. ****p < 0.0001; *p < 0.05.

We performed RNAseq and DEGs on both LKB1mut and LKB1wt cell lines after treatment with MET. As shown in Fig. 2A, AMPK gene expression and AMPK signaling pathway (hsa04152) were significantly upregulated in LKB1mut cells treated with MET compared to untreated cells. Interestingly, treatment with MET in LKB1mut cells induced a significant increase of IFNβ, CCL5, CXCL10 and IRF3 related to the Cytosolic DNA-sensing pathway (map04623) (Fig. 2B). On the contrary, MET treatment did not show the same effect on AMPK signaling pathway and Cytosolic DNA-sensing pathway (Fig. 2C-D) in LKB1wt cells, thus indicating that MET activates AMPK and cytosolic DNA sensing pathways in LKB1mut NSCLC cells.

To further confirm the activation of the DNA sensing pathways induced by metformin treatment in LKB1 mutant NSCLC cells, we conducted IF staining to assess the subcellular translocation of activated p-IRF3 to the nucleus, where it promotes the transcription of IFN genes. As illustrated in Fig. 2E, we observed a significant translocation of p-IRF3 to the nuclear compartment following metformin treatment in H460 cells compared to the CTR. In contrast, no significant translocation of p-IRF3 was detected in H1299 cells, as shown in Supplementary Fig. S2. Overall, our results confirm that metformin induces cGAS-STING activation to a greater extent in LKB1 mutant NSCLC cells.

### Effect of metformin on immune cells derived from naive NSCLC patients

**Fig. 3:**
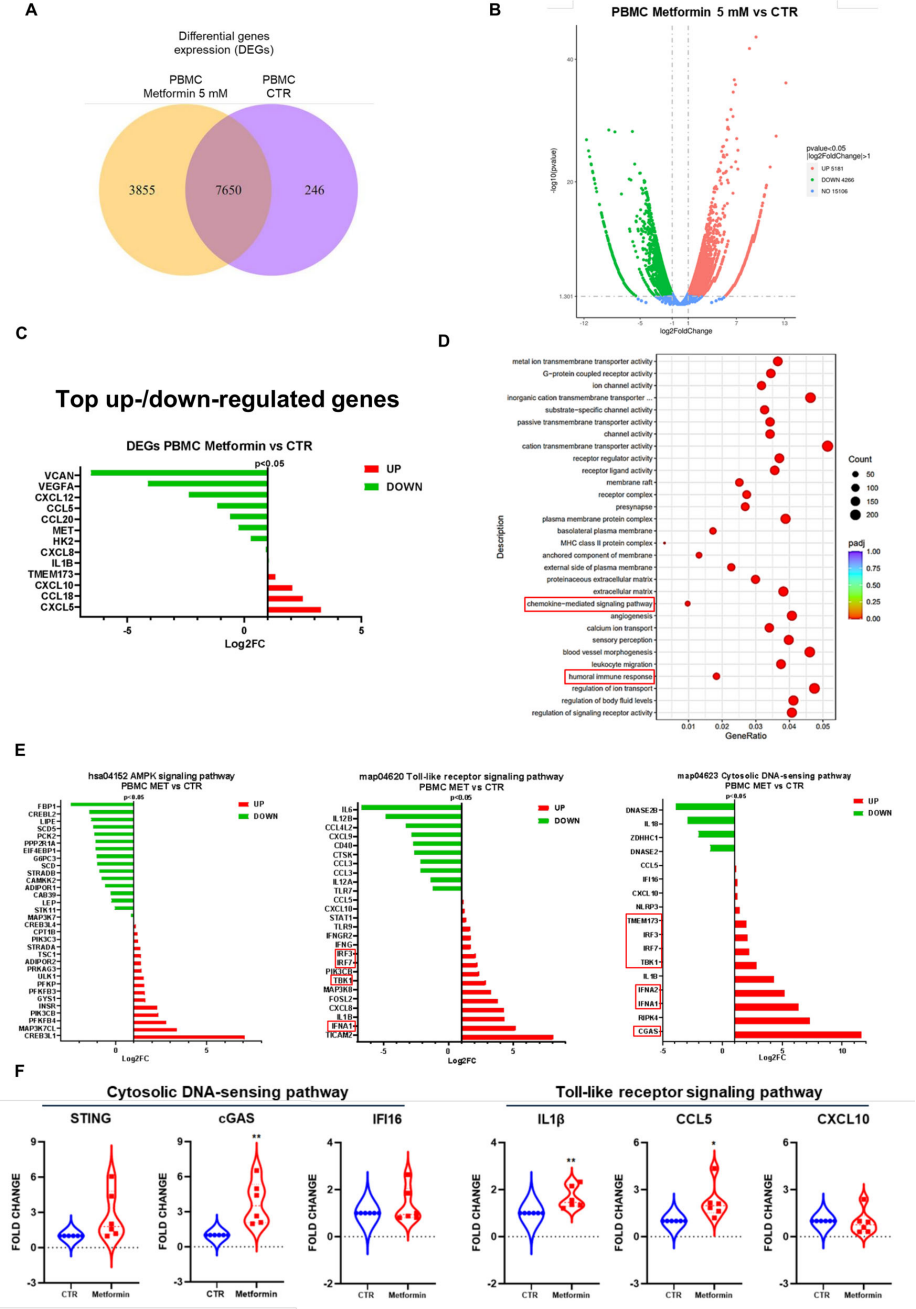
Effect of treatment with metformin in NSCLC patients-derived PBMCs. **A** DEGs in MET-treated NSCLC patient-derived PBMC vs CTR. **B** Volcano plot of DEGs analysis in PBMCs treated with MET compared to CTR. Data are presented as Log2FC values, green and red listed genes *p* value is < 0.05. **C** Most significant DEGs in NSCLC patients-derived PBMCs vs CTR. The red and green bars represent the upregulated genes and downregulated genes, respectively for the differential expression of the selected pathways based on their RNA-seq expression. Data are presented as Log2FC values, green and red listed genes *p* value is < 0.05. **D** Dot plot of DEG showing the top significant enriched biological process (BP) related to MET treated PBMCs vs CTR. Gene Ratio is defined as the ratio between intersection size and query size. The dot sizes represent enrichment abundance while the colors represent the adjusted *p* values (padj) (blue to red). **E** Results of KEGG pathway enrichment analysis in PBMCs treated with MET compared to CTR. The red and green bars represent the upregulated genes and downregulated genes, respectively for the differential expression of the selected pathways based on their RNA-seq expression. Data are presented as Log2FC values, all the listed genes *p* value is < 0.05. **F** Real-time PCR analysis of in vitro STING, c-GAS, IFI16, IL1β, CXCL10 and CCL5 mRNA expression in patient-derived PBMCs. Changes in mRNA levels were normalized to the expression of housekeeping genes (18S). Data are expressed as means ± SEM derived from *n* = 2 technically calculated by the comparative method 2 − ∆∆Ct. One-tailed unpaired Student’s t-test with CI = 95%. Statistical significance: **p < 0.01; *p < 0.05.

Given the broad antitumor effects mediated by MET treatment on immune cells in different solid tumors, especially increasing tumor infiltrating lymphocyte levels and upregulating T-cell inflamed expression profile^12^, we characterized the effect of MET towards immune cells derived from naive NSCLC patients be RNA-seq and DEGs (Fig. 3A). In particular, as shown in Fig. 3B, we found 5181 upregulated genes and 4266 downregulated genes in PBMCs treated with MET compared to CTR. The top down regulated genes (*P* < 0.05) were VCAN (Log2FC = −6,54391), VEGFA (Log2FC = −4,11021), CXCL12 (Log2FC = −2,36826), CCL5 (Log2FC = −1,152) and CCL20 (Log2FC = −0,59993) (Fig. 3C). VCAN and VEGF play a role in the regulation of cell motility, growth and differentiation. CXCL12 and CCL5 act as chemoattractant active on T-lymphocytes and monocytes. CCL20 is responsible for the chemotaxis of effector/memory T-cells and B-cells [18]. Among the most significant upregulated genes in MET treated PBMCs compared to CTR we found CXCL5 (Log2FC = 3,284284), CCL18 (Log2FC = 2,510331), CXCL10 (Log2FC = 2,063503), and STING-gene TMEM173 (Log2FC = 1,333424). CXCL5 and CCL18 are mainly involved in the chemotactic activity of neutrophil and lymphocytes [19]. CXCL10 is known to play pleiotropic effects, including stimulation of monocytes, natural killer and T-cell migration, and modulation of adhesion molecule expression [20]. It is also a key regulator of immune response to viral infections [21] and a key cGAS-STING downstream chemokine involved in immune response [9]. Finally, innate immune activation through STING gene upregulated transcription (TMEM173) indicates that PBMCs treated with MET increase features of innate immune response.

We then performed Gene Ontology (GO) enrichment analysis of NSCLC patients-isolated PBMCs treated with MET compared to untreated CTR. Figure 3D shows that MET treatment induced a positive regulation of gene sets related to transmembrane transporters and ion channels activities along with chemokines mediated signaling pathway and humoral immune response in PBMCs from NSCLC patients compared to CTR. Transmembrane transport of ions represent an essential feature for the maintenance of subcellular organelle integrity and homeostasis in immune cells [22] and favors the release of inflammatory cytokines [23].

We therefore analyzed DEGs results from RNA-seq data in terms of AMPK, Toll-like receptor signaling and cytosolic DNA-sensing pathways enrichment in NSCLC patients-derived PBMCs after treatment with MET compared to untreated CTR (Fig. 3E). Among the AMPK signaling pathway (hsa04152), we found that the endoplasmic reticulum (ER) protein CAMP Responsive Element Binding Protein 3 Like 1 (CREB2L1) was the most upregulated gene along with the TGF-Beta Activated Kinase MAP3K7CL, thus indicating an increase of immune activation in PBMCs. Regarding the Toll-like receptor signaling pathway (map04620) the most enriched gene transcription was the NF-Kappa-B-Activating Protein 502 (TICAM2), known for its role in facilitating the downstream signaling leading to type I interferon induction, along with enrichment of IFNA, IL1B and CXCL8. Finally, the analysis of cytosolic DNA-sensing pathway (map04623) showed that the most upregulated gene was cGAS as well as enrichment of RIPK4 gene which is known for its role in NF-kappa-B activation. We further confirmed these findings by RT-qPCR by analyzing the expression of cytosolic DNA-sensing pathway and Toll-like receptor signaling pathway in PBMCs isolated from NSCLC patients (Fig. 3F). Interestingly, we found a significant increase of cGAS (*p* < 0.01), IL-1β (*p* < 0.01) and CCL5 (*p* < 0.05) in NSCLC patients-derived PBMCs after treatment with MET compared to untreated CTR. Overall, these data suggest a positive pro-immune effect induced by MET treatment on PBMCs of NSCLC patients.

### Effect of metformin and anti-PD-L1 combination on lymphocytes antitumor activity towards NSCLC cells

**Fig. 4:**
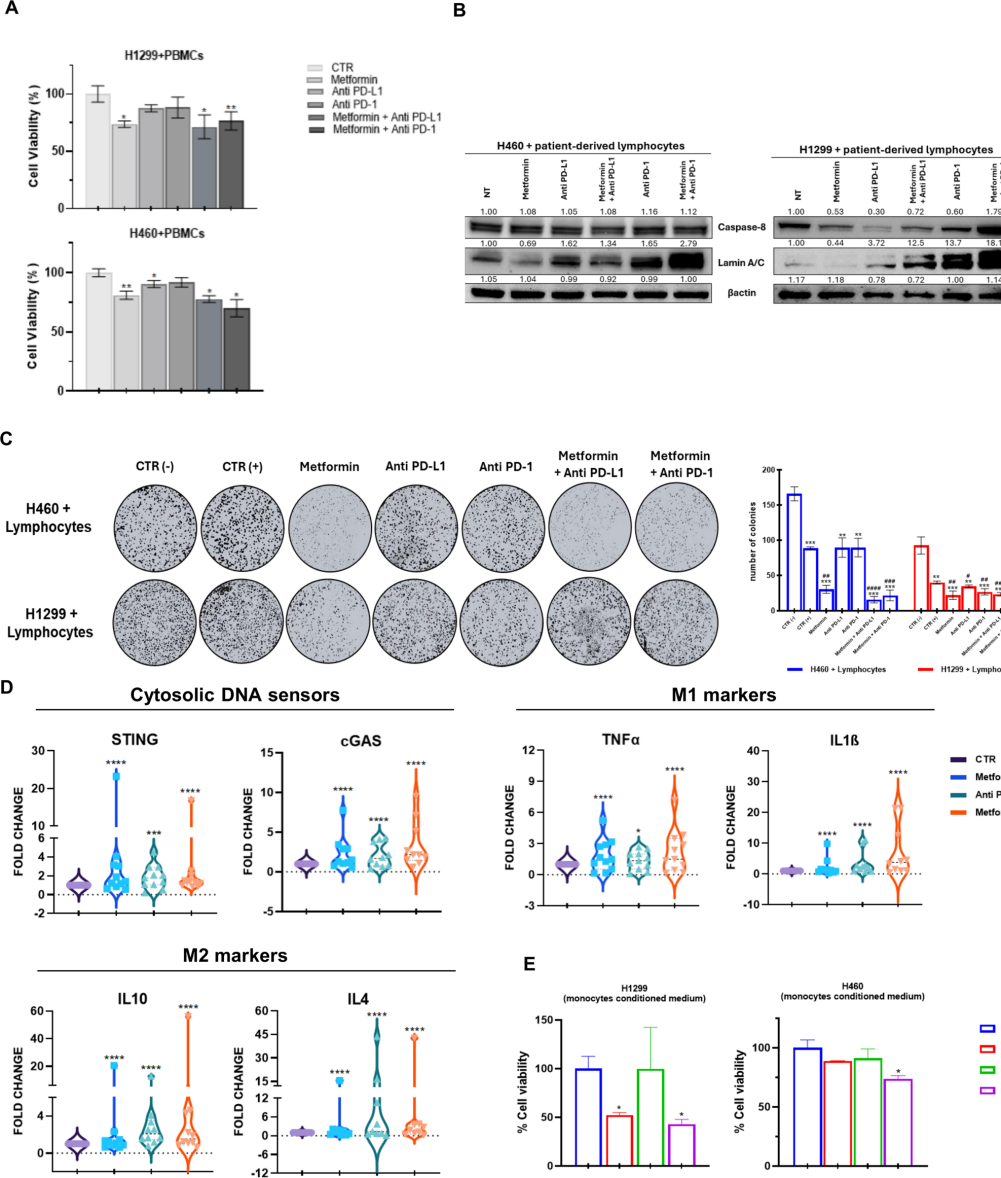
Effect of metformin and anti-PD-L1 combination on lymphocytes antitumor activity towards NSCLC cells. **A** Cell viability assay of LKB1wt (H1299) and LKB1mut (H460) NSCLC cells exposed to NSCLC patient-derived PBMCs pre-treated with metformin (MET) and anti-PD-L1 for 48 h. Data are expressed as mean ± SD. At least three independent experiments were performed. **B** Western blotting of H460 and H1299 NSCLC cell lines after 48 h of co-culture with NSCLC patient-derived PBMCs. β-actin was used to ensure equal loading. **C** H460 and H1299 NSCLC cell lines were seeded in a 12-well plate (3000 cells/well). The cells were preincubated for 48 h with NSCLC patient-derived PBMCs pre-treated with drugs for 5 days. Afterward, the cells were fixed and stained with 0.5% crystal violet solution. Images of representative wells were scanned and are shown. Bar plots of colony numbers for H1299 NSCLC cells are shown in blue, while H460 NSCLC cells are shown in red. Results are expressed as the mean of three technical replicates ± SD. **D** Real-time PCR analysis of in vitro STING, c-GAS, CXCL10 and CCL5 mRNA expression in patient-derived monocytes (*n* = 11). Changes in mRNA levels were normalized to the expression of housekeeping genes (18S). Data are expressed as means ± SEM derived from *n* = 2 technically calculated by the comparative method 2 − ∆∆Ct. One-tailed unpaired Student’s t-test with CI = 95%. **E** Cell viability assay of NSCLC cell lines cultured with NSCLC patient-isolated monocytes treated with MET and/or anti-PD-L1 for 48 h conditioned media. Each experiment was performed in triplicate. Data are expressed as mean ± SD. ****p < 0.0001; ***p < 0.001; **p < 0.01; *p < 0.05.

We then investigated the effect of MET treatment combined with anti-PD-L1 drugs in inducing a functional anti-tumor immune response in immune cells towards NSCLC cells based on their LKB1 status. As we reported in our previous studies [24], H460 and H1299 cell lines show a percentage of PD-L1 of 85%. In Fig. 4A, we assessed the viability of LKB1mut and LKB1wt NSCLC cells co-cultured with NSCLC patients-derived PBMCs pre-treated with MET alone or in combination with an anti-PD-L1. Interestingly, we found that LKB1mut NSCLC cell line H460 showed a higher reduction of cell viability when co-cultured with PBMCs treated with MET alone and combined with anti-PD-L1 compared to LKB1wt cells (Fig. 4A). After co-culture with immune cells, we analyzed the protein expression of NSCLC cells H1299 and H460 in terms of cell cycle and apoptosis markers expression to further investigate the mechanism of cell death induced by MET-activated immune cells. We found that PBMCs treated with MET alone were not able to induce in vitro cell death in NSCLC cell lines, whereas the combination of MET with anti-PD-1/PD-L1 activated immune cells-mediated cytotoxicity through Caspase-8 increase in H1299 and strong Lamin A/C upregulation (Fig. 4B).

We also investigated the effects of combining MET with immunotherapeutic agents on the induction of anti-tumor activity in immune cells. As illustrated in Fig. 4C-D, a modest decrease in colony numbers was observed in H460 cells cultured with PBMCs compared to the control group. Treatment with MET alone or in combination with anti-PD-L1 significantly diminished the colony-forming capacity as well as the immune cell response towards these LKB1-mutant cells. This finding suggests that the combination of MET and immunotherapy can influence the colony formation ability of LKB1-mutant tumors activating immune cells to exhibit anti-tumor activity. Furthermore, anti-PD-1/PD-L1 did not affect colony formation, reinforcing the notion that LKB1-mutant cells exhibit intrinsic resistance to immunotherapy. Interestingly, PBMCs co-cultured with H1299 cells significantly impacted colony formation compared to the control. The application of MET, anti-PD-L1, anti-PD-1, and their combinations resulted in a substantial reduction in colony numbers within H1299 cells. These results indicate that the addition of MET to immunotherapeutic regimens effectively promotes an immune-mediated anti-tumor response against both LKB1-mutant and LKB1-wild type cells. Overall, the co-culture findings suggest that MET is capable of activating anti-tumor immune cell functions, leading to in vitro cytotoxicity, apoptosis, and decreased long-term tumor survival, independent of LKB1 mutational status. This implies that patients exhibiting poor responses to immunotherapy may benefit from the incorporation of MET into their treatment regimen.

Monocytes play a central role in mediating anti-tumor functions of immune responses [25], so we analyzed the effect of MET on PBMC-isolated monocytes. PBMCs from naive NSCLC (*n* = 11) were collected and subsequently monocytes were isolated in culture by adherence. After treatment with MET and/or anti-PD-L1 for 48 h we analyzed the mRNA expression of the cGAS-STING innate immune pathway. Interestingly, both STING and cGAS were significantly upregulated after treatment with MET alone or combined with anti-PD-L1 in NSCLC patients-derived monocytes (*p* < 0.0001). Then we characterized by RT-PCR the anti-tumoral M1 and pro-tumoral M2 phenotypically distinct subpopulations (Fig. 4D) [26]. As shown in Fig. 4D, we found that combination treatment of MET and atezolizumab significantly increased the M1 markers IL-1β, and TNF-α as compared to control and with a higher fold increase compared to the M2 markers modulation.

Finally, in Fig. 4E, we evaluated the capacity of pre-treated monocytes to mediate an in vitro anti-tumor effect through co-culture with NSCLC cells. Notably, the conditioned media from MET-treated monocytes significantly reduced cell viability in LKB1-wild type cells. In contrast, the combination of MET with immunotherapeutic agents demonstrated efficacy against both cell lines, regardless of LKB1 mutational status. This further corroborates the benefits of utilizing MET in conjunction with immunotherapy.

### Effect of metformin and anti-PD-L1 combination on immune cells antitumor activity towards NSCLC tumor spheroids and patients-derived 3D tumor organoids

**Fig. 5:**
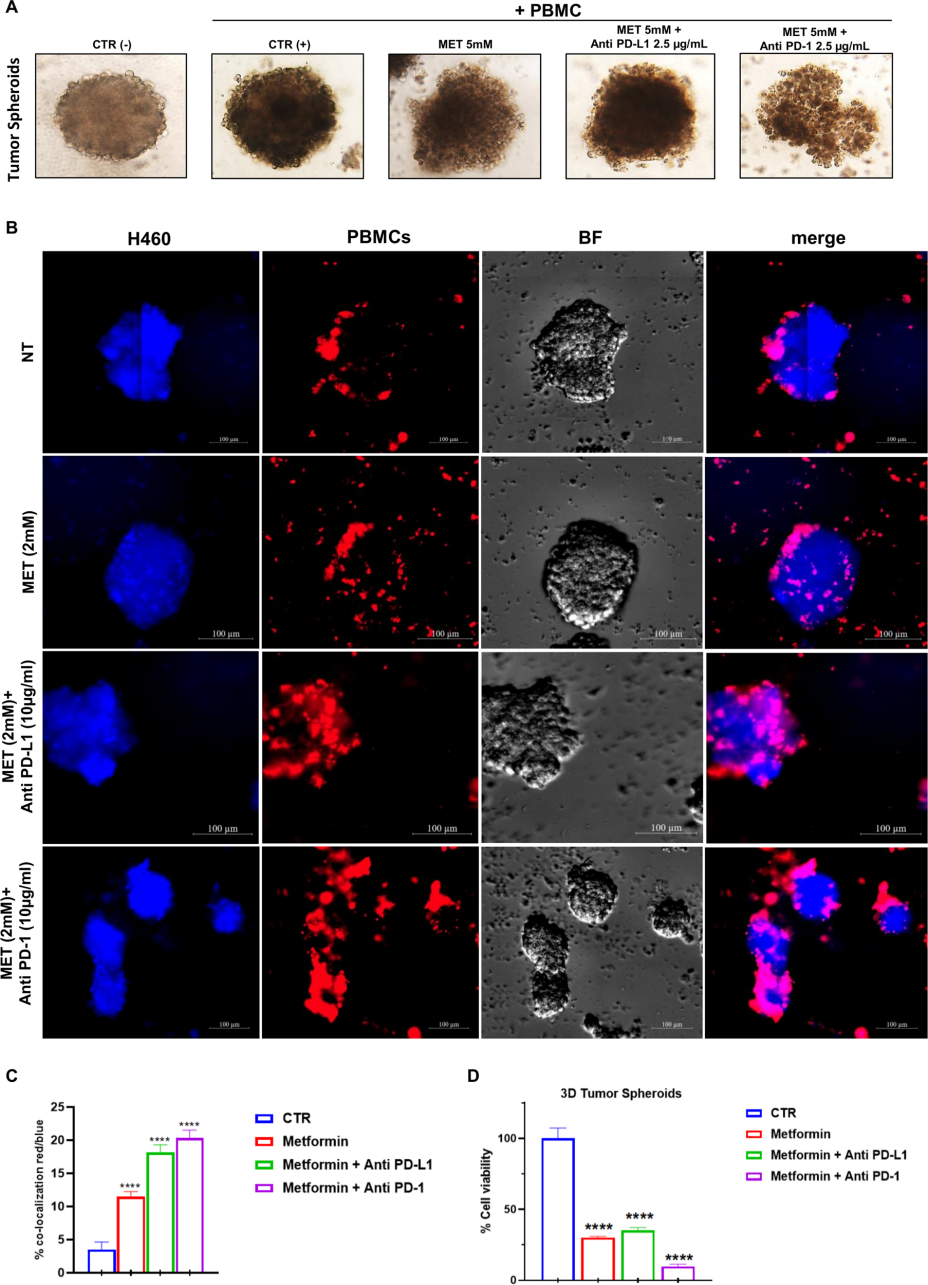
Effect of metformin and anti-PD-L1 combination on immune cells antitumor activity towards NSCLC LKB1mut 3D tumor spheroids. **A** Bright-field images of LKB1mut H460 spheroids plated in culture 6 days after plating and treated with MET and anti-PD-L1 or anti-PD-1. Scale bars, 100 mm. **B** Representative IF images showing the infiltration ability of immune cells in 3D tumor spheroids of H460 cells. 3D tumor spheroids were blue stained, and the NSCLC patient-derived PBMCs were red stained. The PBMCs were treated with MET and anti-PD-L1 or anti-PD-1 for 48 h and added to 3D spheroids to start the co-culture. The co-localization of NSCLC cells and PBMCs is reported under the merge column. Scale bar 100 µm. **C** Evaluation of the red/blue intensity ratio as a quantitative indication of the colocalization rate of LC cells and PBMC. ****p < 0.0001. **D** Cell viability assay of PDTOs co-cultured for 48 h with MET, anti-PD-L1 or anti-PD-1 pre-treated NSCLC-patient derived PBMCs. Data are expressed as mean of three independent experiments ± SD. ****p < 0.0001.

We functionally validated our in vitro findings regarding the pro-immune effects of MET on PBMCs derived from NSCLC patients, utilizing 3D NSCLC cold tumor spheroids derived from LKB1-mutant cells. Following the formation of tumor spheroids, we established a 3D culture comprising both tumor spheroids and immune cells derived from NSCLC patients, and we monitored the development of the tridimensional structure of the spheroids generated from LKB1-mutant NSCLC H460 cells over time (Fig. 5A). After 48 h of co-culture, we assessed the infiltration capacity of immune cells (Red) into the 3D tumors (Blue) (Fig. 5B). Our results indicated that treatment with MET, both alone and in combination with anti-PD-1 or anti-PD-L1 agents, significantly enhanced the co-localization of immune cells within the tumor spheroids (Fig. 5C). Concurrently, there was a notable reduction in the viability of the tumor spheroids (Fig. 5D). This model provides a functional in vitro framework for predicting the pro-immune effects induced by MET on LKB1-mutant tumors. Moreover, these findings suggest that MET has the potential to convert cold tumors into inflamed ones.

**Fig. 6:**
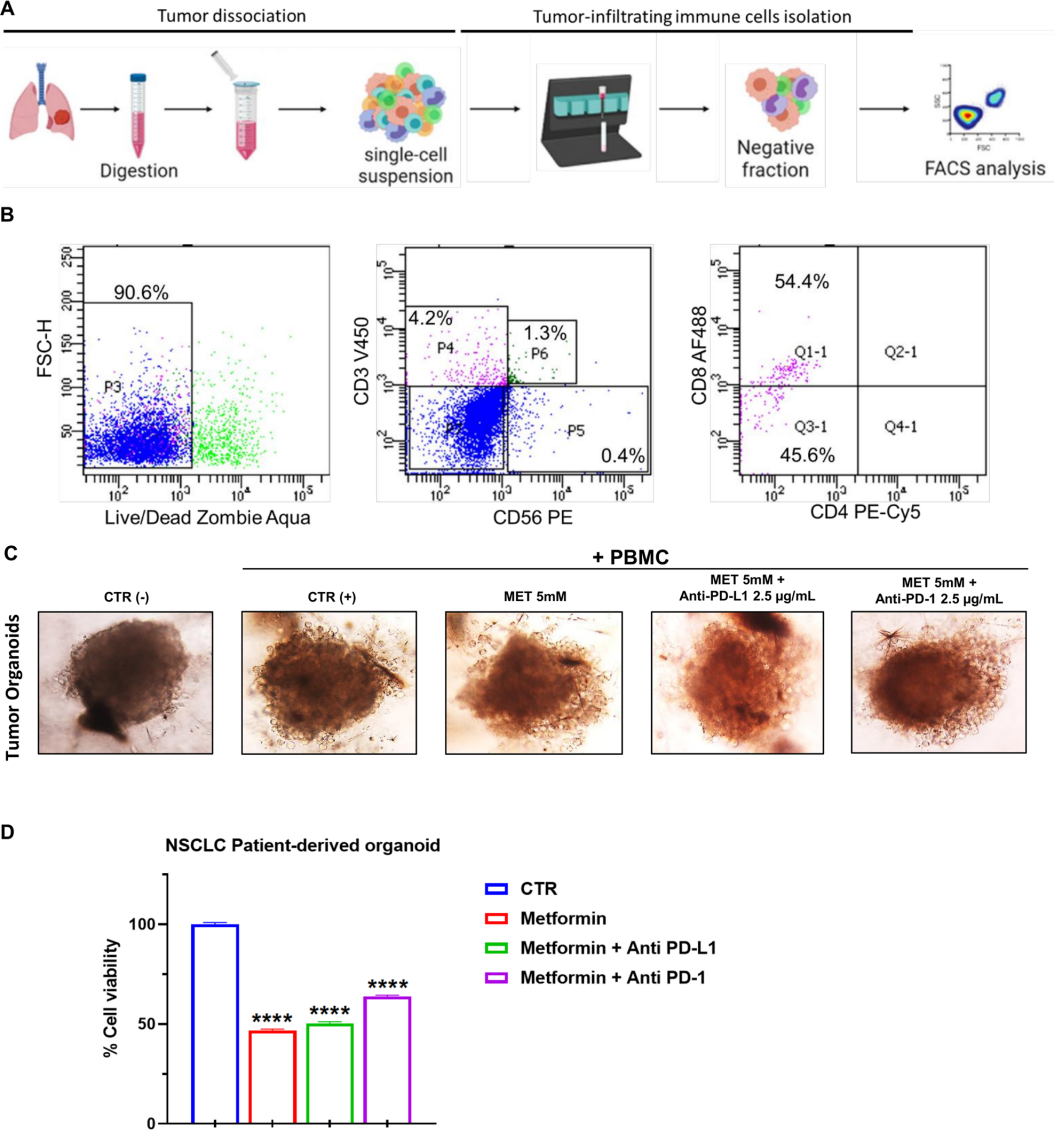
Effect of metformin and anti-PD-L1 combination on immune cells antitumor activity towards NSCLC patient-derived tumor organoid. **A** A graphical summary of the tumor-infiltrating isolation method step-by-step. The graphical scheme was produced by the authors using the free BioRender platform. **B** Flow cytometry plots for the analysis of live lymphocyte subsets in NSCLC tumor tissue. Cells were first gated into CD3+ or CD56 + , then CD4 + T cells (CD3 + CD56 − CD4 + CD8 − ) and CD8 + T cells (CD3 + CD56 − CD4 − CD8 + ) were identified. Numbers adjacent to the outlined areas indicate the percentage of cells in each sample. **C** Bright-field images of NSCLC patient-derived cancer organoids plated in disc culture 6 days after plating. Scale bars, 100 mm. **D** Cell viability assay of NSCLC PDTOs treated with MET alone or combined with anti-PD-1 or anti-PD-L1 for 48 h. Data are expressed as mean of three independent experiments ± SD. ****p < 0.0001.

Finally, we validated our in vitro findings on the pro-immune effect of MET on NSCLC patients derived PBMCs in an ex vivo 3D tumor established from NSCLC biopsy. PD-L1 expression levels of tumor tissue biopsy from NSCLC patients used for 3D tumor establishment is reported in Fig. S2B. After tissue biopsy digestion, we isolated tumor-infiltrating lymphocytes (TILs) by magnetic beads conjugation (Fig. 6A). Then we performed flow cytometry analysis to characterize and quantify the % of TILs. As shown in Fig. 5A, 90.6% of live TILs after isolation, with 4.2% being CD3+ cells and 0.4% being CD56+ cells. Among the CD3+ gated cells, the majority of cells were cytotoxic T lymphocytes (CD8 + ). In Fig. 6B we summarized the main subpopulation of TILs isolated from NSCLC patient biopsy. Thereafter, we established a 3D culture of biopsy isolated tumor and immune cells, and we monitored the tridimensional structure formation over time (Fig. 6C). Once the organoids culture was established, after several passages the immune cells could be depleted so we rinsed PDTOs with autologous PBMCs pre-treated with MET combined with anti-PD1/PD-L1 drugs. After 48 h of co-culture, we tested cell viability of both tumors organoid and spheroid. As shown in Fig. 6D, MET alone or combined with anti-PD-L1 was able to induce a significant reduction in cell viability of PDTOs, ultimately confirming the translational potential of combination of metformin and anti-PD-1/PD-L1 agents.

## Discussion

In recent years, immunotherapy has become one of the major therapy options for advanced NSCLC patients that has greatly benefited from this approach. According to current selection criteria, however, not all patients achieve a durable response to immune checkpoint inhibitors [27]. In this context, the development of either primary or secondary resistance to immunotherapy highlights a need for alternative MET to modulate anti-tumor immune functions. Recently, it has been proved that MET combined with PD-1 inhibitor enhanced anti-tumor efficacy in STK11 mutant NSCLC through inhibition of STING ubiquitination [28], suggesting a positive pro-immune effect of MET in patients with poor response to immunotherapy. Previous clinical trials have shown a clinical benefit of the addition of MET in NSCLC patients undergoing immunotherapy [29, 30], but the small sample sizes and lack of evaluation of LKB1 mutations may have limited their conclusions.

Our study employed a diverse array of in vitro and ex vivo models, including NSCLC cells, patient-derived immune cells, 2D and 3D tumor spheroid co-cultures, and PDTOs. We demonstrated that combinations of MET with anti-PD-1/PD-L1 effectively stimulate an anti-tumor immune response by activating key cytosolic DNA sensors and enhancing pro-immune characteristics, particularly in LKB1 mutant NSCLC tumors. These results provide new insights into the pro-immune effects of MET, suggesting its potential to reactivate immune responses in patients with poor immunotherapy outcomes. We propose that MET-treated immune cells may increase chemokine secretion, aiding the recruitment of additional immune cells to the tumor microenvironment and overcoming the immunosuppressive barriers present in LKB1mut tumors.

However, several limitations must be noted. The results from cellular and organoid models may not fully capture the complexities of the in vivo tumor microenvironment. Additionally, patient responses to immunotherapies can vary significantly due to intrinsic heterogeneity, complicating predictions regarding the effectiveness of MET combinations across broader populations. Longitudinal studies are essential to assess both short-term efficacy and the durability of immune responses. These considerations highlight the need for further research to validate our findings and refine therapeutic strategies for NSCLC.

In conclusion, our study indicates that the combination of MET with anti-PD-1/PD-L1 therapies may serve as a promising therapeutic strategy to augment the innate immune response in patients diagnosed with NSCLC, especially for those harboring LKB1 mutations which tend to be poorly immune responsive By leveraging the unique mechanisms of action associated with MET, which appears to modulate key signaling pathways and promote pro-immune features, we provide compelling evidence for its potential role in reactivating immune responses in a subset of NSCLC patients.

## Supplementary information

Supplemental Material

uncropped gels

## Data Availability

The majority of data related to presented results are included in the materials and methods section of the paper. RNA-seq raw data were generated at Novogene, Inc. and are available from the corresponding authors on request.
