# Supplementary material for: Addition of metformin to anti-PD-1/PD-L1 drugs activates anti-tumor immune response in peripheral immune cells of NSCLC patients
Source: Cell Death Dis. 2025 Apr 13;16(1):286. doi: 10.1038/s41419-025-07636-7 (PMC11993597; doi:10.1038/s41419-025-07636-7)
Supplement: Supplementary file 1 — Supplemental Material [file 41419_2025_7636_MOESM1_ESM.docx]

**Supplementary Fig. S1**


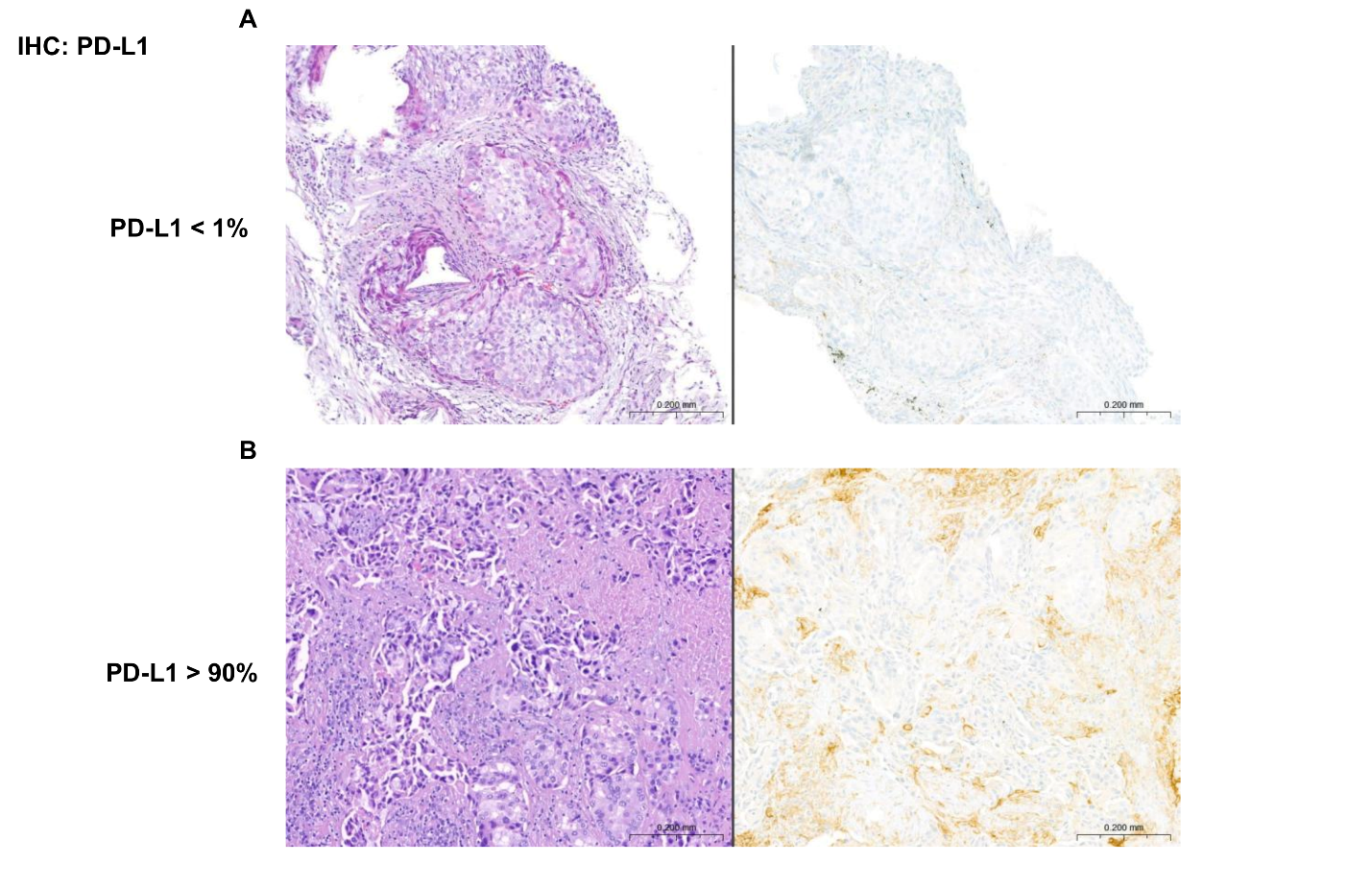


**(A)** left panel - Morphological picture of lung infiltration by some nests of poorly differentiated squamous cell carcinoma cells. (H&E staining, original magnification: 10x). right panel - Immunostaining for PD-L1 showing complete lack of PD-L1 membranous expression by neoplastic cells of squamous cell carcinoma of the lung (TPS: <1%) (original magnification: 10x). **(B)** left panel- Morphological picture of stromal invasion by non mucinous adenocarcinoma of the lung. Neoplastic nets and tumoral necrosis can be observed (H&E staining, original magnification: 5x). right panel - Immunostaining for PD-L1 showing very occasional membranous expression by the neoplastic cells (TPS: 2%) (original magnification: 5x).

**Supplementary Fig. S2**

**
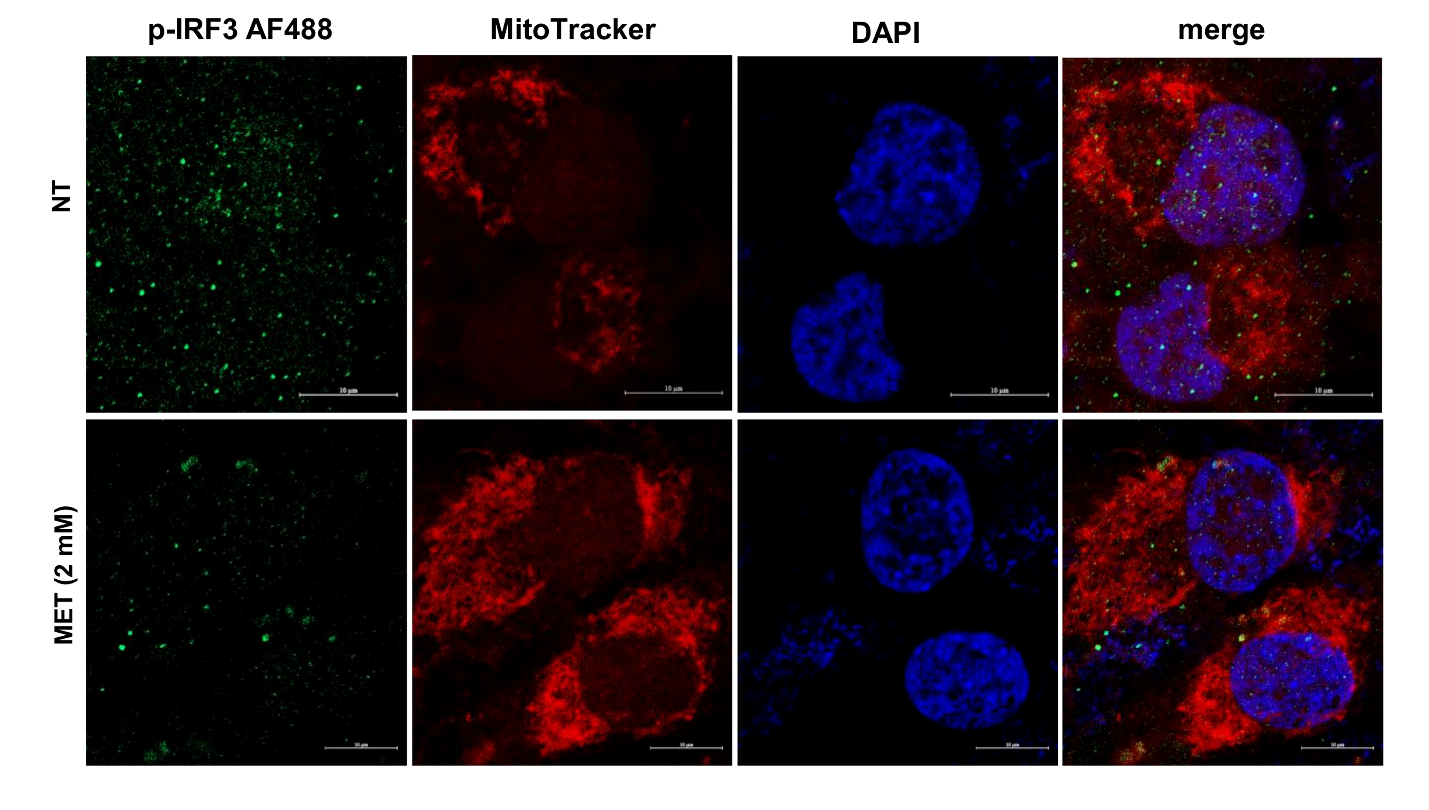
**

Representative immunofluorescence images and MFI quantification showing p-IRF3 (green), MitoTracker (red) and DAPI (blue) in H1299 cells treated with MET 2 mM (scale bar: 10 μm, Magnification 63X).

**Supplementary Table 1. List of primer sequences used for qRT-PCR**

| **Gene** | **Forward Sequence** | **Reverse Sequence** |
| --- | --- | --- |
| *18S* | *5′-CGCCGCTAGAGGTGAAATTC-3′* | *3′-CTTTCGCTCTGGTCCGTCTT-5′* |
| *STING* | *5’-CAGGCACTGAACATCCTCCT-3’* | *3’-ATATACAGCCGCTGGCTCAC-5’* |
| *cGAS* | *5’-CTCCACGAAGCCAAGACCTC-3’* | *3’-GCGGCTGAGCTTCAACTTCT-5’* |
| *TNFα* | *5’-TCTGTCTGCTGCACTTTGGAGTGA-3’* | *3’-TTGAGGGTTTGCTACAACATGGGC-5’* |
| *IL10* | *5’-TCTCCGAGATGCCTTCAGCAGA-3’* | *3’-TCAGACAAGGCTTGGCAACCCA-5’* |
| *IL4* | *5’-CCGTAACAGACATCTTTGCTGCC-3’* | *3’-GAGTGTCCTTCTCATGGTGGCT-5’* |
| *IL1β* | *5’-CCACAGACCTTCCAGGAGAATG-3’* | *3’-GTGCAGTTCAGTGATCGTACAGG-5’* |

**Supplementary methods**

**RNA sequencing method procedure -** 1 µg of purified total RNA was used for library preparation. mRNA was purified from the total RNA using poly-T oligo-attached magnetic beads. After fragmentation, the first strand cDNA was synthesized using random hexamer primers, followed by second strand cDNA synthesis using either dUTP for the directional library or dTTP for the non-directional library. The non-directional library was prepared after end repair, A-tailing, adapter ligation, size selection, amplification, and purification. The directional library was prepared after end repair, A-tailing, adapter ligation, size selection, USER enzyme digestion, amplification, and purification. The library was checked with Qubit and real-time PCR for quantification and a bioanalyzer for size distribution detection. Quantified libraries were pooled and sequenced on an Illumina platform (NovaSeq X Plus Series PE150). Raw data in FASTQ format were first cleaned using fastp. Clean reads were obtained by removing reads containing adapters, poly-N and low-quality reads from raw data. The reference genome hg38 from the NCBI website was used, and paired-end cleaned reads of the samples were mapped. The index of the reference genome was built using Hisat2 v2.0.5 and paired-end clean reads were aligned to the reference genome using Hisat2 v2.0.5. The mapped reads of each sample were assembled by StringTie (v1.3.3b) in a reference-based approach. Read counts were performed using FeatureCounts v1.5.0-p3, and differential expression analysis was performed using R with the DESeq2 package (v1.20.0). The resulting P-values were adjusted using Benjamini and Hochberg's approach for controlling the false discovery rate. Genes with an adjusted P-value <=0.05 found by DESeq2 were assigned as differentially expressed.

**PDTO culture medium composition -** Complete medium composition was as follows: 1X Antibiotic-antimycotic, 1X Glutamine, 1X Hepes, 1X B27, 1.25 mM N-acetyl L-cysteine, 10 mM nicotinamide, 100 ng/ml hNoggin, 500 nM A-83–01, 10 μM SB202190 monohydrochloridehydrate, 500 ng/mL hRSPO1, 20 ng/mL hFGF10, 10 μM Y-27632 dihydrochloride. A portion of all PDTOs was cryopreserved and stored to establish a comprehensive living organoid bank, which will sustain further investigational projects.
