# Supplementary figures and images for: Addition of metformin to anti-PD-1/PD-L1 drugs activates anti-tumor immune response in peripheral immune cells of NSCLC patients
Source: Cell Death Dis. 2025 Apr 13;16(1):286. doi: 10.1038/s41419-025-07636-7 (PMC11993597; doi:10.1038/s41419-025-07636-7)

Uncropped gel for western blot shown in Figure 4B

H460

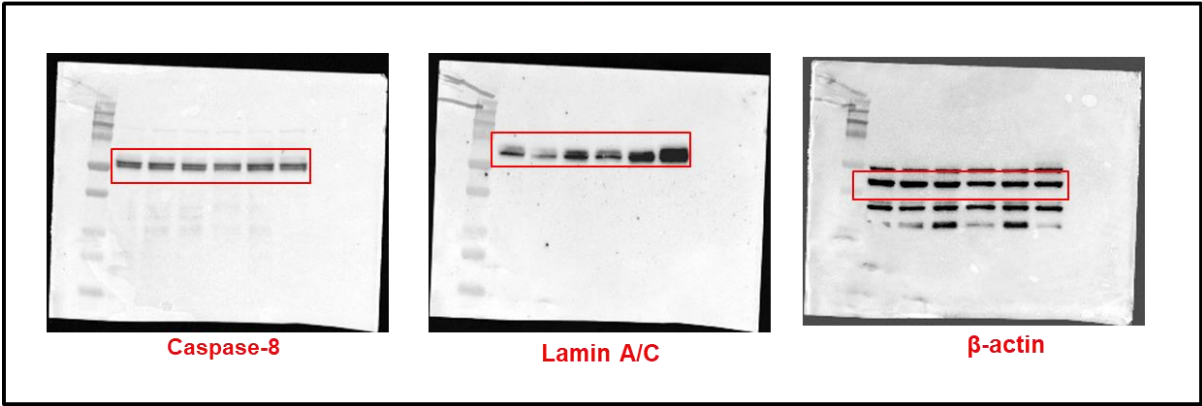

H1299

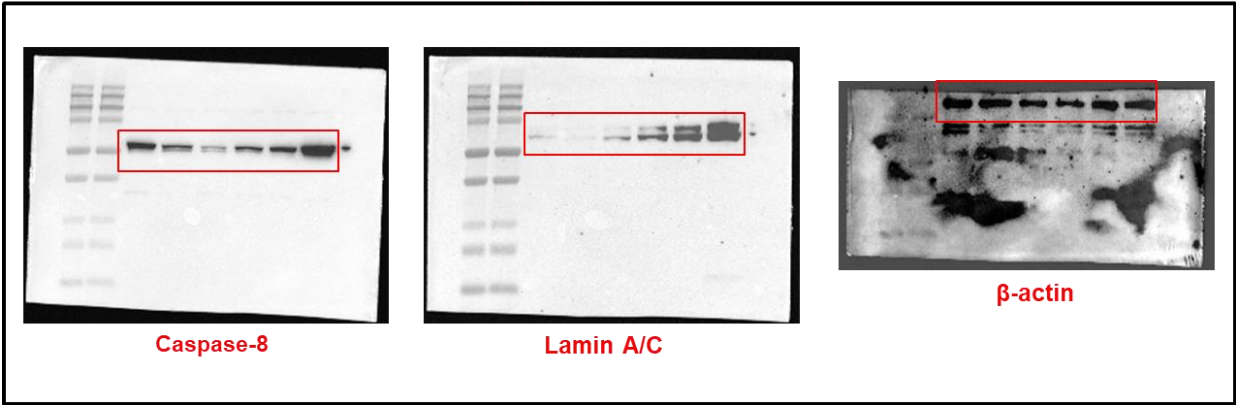

Supplement: Supplementary file 2 — uncropped gels [file 41419_2025_7636_MOESM2_ESM.pdf]
